# “I feel so small and big at the same time”—exploring body experience and binge eating disorder following inpatient treatment: a qualitative study

**DOI:** 10.3389/fpsyg.2024.1432011

**Published:** 2024-10-14

**Authors:** Helene T. Olsen, Sunniva B. Vangen, Line Indrevoll Stänicke, KariAnne Vrabel

**Affiliations:** ^1^Department of Psychology, University of Oslo, Oslo, Norway; ^2^Lovisenberg Hospital, Nic Waals Institute, Oslo, Norway; ^3^Research Institute of Modum Bad, Vikersund, Norway

**Keywords:** binge eating, eating disorder, qualitative, treatment experience, body experience

## Abstract

**Background:**

Limited research into binge eating disorder (BED), a low treatment rate, and a lack of treatment rights, reflects a marginalized disorder in society and a treatment context.

**Aim:**

The aim of this study was to gain a deeper understanding of the psychopathology of BED, by exploring the patients’ meanings related to the disorder and the role of the body in the treatment of BED.

**Method:**

Qualitative methodology using a reflexive thematic analysis. The data material is based on semi-structured in-depth interviews with six cis-gendered women who had previously completed an inpatient treatment program for BED at a department for EDs and met the criteria for 307.51 Binge Eating Disorder in DSM-5.

**Results:**

The qualitative analysis rendered two meta-themes comprising six themes and 12 subthemes. The initial meta-theme was “Relational challenges and feelings could not be talked about at home.” The secondary meta-theme was “Body contempt disturbs the experience of self and others” and the third was “Their body has not been a theme in previous treatment.” This categorization depicts a narrative of the disorder in terms of development, current condition, and experiences with prior treatment.

**Discussion:**

The findings indicate that shame and disgust are central to playing pivotal roles in the embodiment of BED, highlighting the significance of addressing one’s relationship with their body for achieving recovery and preventing an experienced repetition of the perceived rejection as a child.

## 1 Introduction

Binge eating disorder (BED) is a relatively new diagnosis and was included in the DSM-5 [American Psychological Association (APA)] in 2013 and the revised edition of ICD-11 (World Health Organization, 2019). The disorder is categorized as an eating disorder (ED) and is defined as overeating unusually large amounts of food, combined with experiencing a lack of control when eating. This is also a central feature of Bulimia Nervosa and subtypes of Anorexia Nervosa, whereas with BED there is an absence of compensatory strategies for the energy intake (American Psychiatric Association, 2013). BED is the most frequent ED, with an estimated lifetime prevalence of 1.5–3.5% among women and 0.3–2% among men (Watson et al., 2018; Keski-Rahkonen, 2021). The disorder is associated with significant psychological and physical comorbidity (Bulik et al., 2002; Keski-Rahkonen, 2021). It influences psychological, physical, and social functioning, and is therefore defined as a public health problem (Austin, 2012). However, access to treatment is highly limited, and studies revealed treatment rates as low as 38% (Kessler et al., 2013).

There is a high incidence of comorbid obesity problems in BED, where 30–40% of the cases of the disorder are associated with obesity and morbid obesity (Kessler et al., 2013). Approximately 30% of people seeking help for weight reduction or waiting for surgical treatment of morbid obesity satisfy the criteria for BED (Götestam and Agras, 1995). Since obesity has been the most prominent (and visible) symptom of the disorder, the research, and treatment focus have often been directed toward the behavioral aspects of the disorder and weight loss goals. The primary emphasis of the research revolves around Randomized Controlled Trials (RCTs) pharmacological interventions. These studies have demonstrated a significant reduction of 50–60% in the frequency of binge eating episodes with the use of Selective Serotonin Reuptake Inhibitors (SSRIs) (Fairburn and Sjøbu, 2014). Additionally, these interventions have demonstrated moderate effects on weight reduction. It is important to note that a substantial proportion of the observed response to drugs can be attributed to placebo effects (Brownley et al., 2007). At the same time, the effect of drugs is known to cease with the completion of the treatment, and the negative side effects of such treatment are significant. This reflects how current weight-reduction treatments have limited functions and lack optimal interventions that lead to sustained weight loss. The patient group often finds itself primarily involved in the somatic aspect of treatment, wherein they are not provided with the psychological treatment they are entitled to.

Although BED is the most frequently occurring ED, studies demonstrate little effect of treatment, and there is very limited research on the experience of having BED (Bremer et al., 2023). Different models have attempted to explain the contributing factors in developing BED, such as media exposure, internalization of a thin ideal, and personality traits such as negative emotionality (Culbert et al., 2015). Several qualitative studies investigating the experience of living with BED explored the experience of guilt, shame, and loss of control as central aspects of the disorder (Perelman et al., 2023; Brownstone et al., 2021; Lord et al., 2018; Salvia et al., 2022; Lewke-Bandara et al., 2020), and acceptance and validation are pointed out as important steps in the recovery process (Lord et al., 2018).

The number of research studies examining the treatment of BED has more than doubled in size in the last decade (Hilbert et al., 2019), mainly consisting of RCTs on the effect of Cognitive Behavioral Therapy (CBT)-E, which is the prevailing treatment in the ED field (CBT) (Fairburn et al., 1999; National Institute for Health and Care Excellence, 2017). Although there is evidence that CBT-E reduces behaviors related to ED (Atwood and Friedman, 2020), studies indicate moderate treatment results (Wilson et al., 2007), with up to 50% of individuals failing to respond to treatment (Delis et al., 2001). Despite increasing recognition of the necessity for further knowledge of the disorder, the field currently lacks studies that specifically investigate the meaning and experiences associated with BED from the perspective of the patients themselves (Brownstone et al., 2021). Our current understanding of the psychological traits that define the disorder is insufficient, and this lack of understanding May have significant clinical implications (Serpell et al., 2020). The weight-related symptoms of the disorder can make it challenging to meet the requirements of the group. It is conceivable that the body shaming they experience due to their body size makes their bodily experience a central concern in the treatment of BED.

Even though there is an increasing awareness of BED in the ED research field through qualitative studies on experiences with treatment programs (Salvia et al., 2023; Rørtveit et al., 2021; Moghimi et al., 2022; Bakland et al., 2019), there remains a notable absence of studies that explore the lived experiences of individuals with BED (Perelman et al., 2023; Brownstone et al., 2021; Lord et al., 2018; Salvia et al., 2022; Lewke-Bandara et al., 2020). According to Bardone-Cone et al. (2019), recovery rates remain below 50%, highlighting the importance of exploring the meaning and experience of living with a BED to reveal factors that may potentially improve treatment and recovery. Argumentatively, we need more knowledge about what the disorder entails and systematic research into the role body experience plays in the treatment of BED and how it affects their experience of self in social relationships. The primary objective of this study is to enhance our understanding of the lived experience of individuals with BED. This will be achieved through a qualitative exploration of patients’ subjective meaning and experience subsequent to inpatient treatment. The secondary aim of this study is to examine the role of bodily experiences in the development and perpetuation of disorder. We aim to address the research gap pertaining to the significance and experiences associated with BED from the patients’ perspective and significance of body experience in the treatment of BED. We would like to raise suggestions on how this could be emphasized in treatment, based on the answers to our research questions: (1) How do patients with BED experience their suffering? (2) How do patients with BED interpret their bodies and their experiences of self in social relationships?

## 2 Method

### 2.1 Design

A qualitative research design allows us to obtain new insights and nuanced descriptions of people’s subjective experiences (McLeod, 2011). The data material contained interviews with people struggling with BED who had a shared experience of inpatient treatment in Norway in a highly specialized ED treatment. The interview topics included descriptions of their disorder and their body experiences through stories of themselves and others. The research was guided by the steps of reflexive thematic analysis (Braun and Clarke, 2006, 2022). We chose an inductive analysis, wherein the themes were semantically based on the explicit experiences of the participants.

### 2.2 Sample and selection

The interviews were conducted from 7 September 2021 to 1 October 2021. The sample consisted of six women between the ages of 25 and 50 who were referred to inpatient, residential treatment for BED at a department of eating disorders. The unit is a psychiatric center that admits patients from various regions of Norway who have undergone previous treatment without achieving desired outcomes. The treatment consisted of 1 week of examination followed by 4–5 weeks at home, and then 8 weeks of intensive psychotherapy, including a week of training at home. Our study employed an integrated treatment approach combining Compassion Focused Therapy (CFT) and CBT to address the multifaceted nature of BED. The CFT component focusses on reducing self-criticism and shame, which are commonly experienced by individuals with eating disorders, by fostering self-compassion and emotional regulation. These techniques included compassionate imagery, developing a compassionate mind, and exercises aimed at enhancing self-kindness and understanding (Goss and Allan, 2010, Goss, 2011; Gilbert, 2014). The CBT component targeted dysfunctional eating behaviors and cognitive distortions associated with BED. This involved cognitive restructuring, self-monitoring, exposure to feared foods, and behavioral experiments designed to challenge and modify maladaptive eating patterns and beliefs (Kelly and Carter, 2015; Kelly et al., 2014). The integration of CFT and CBT aimed to address both the emotional and behavioral dimensions of BED, providing a comprehensive treatment approach to support recovery (Goss and Allan, 2014; Kelly & Tasca, 2016). The weekly program included two individual therapy sessions, one session with a nurse to review the food diary and weigh, and two group sessions consisting of psychoeducation and setting recovery goals with a psychologist. On the last day of the inpatient stay, seven women were invited to participate in this study. All of them initially attended, but one of them withdrew her consent during the project. To be eligible for participation in the treatment, and thus in this study, patients had to (a) meet the DSM-5 criteria for BED, (b) have failed to benefit from any structured psychological treatment, (c) be 18 years of age or older, (d) be able to speak Norwegian, and (e) be capable of providing informed consent. Patients were excluded if they had ongoing serious self-harm or chronic suicidality that could interfere with the interview situation. The interviews were completed within three months after discharge.

### 2.3 Procedure

The interviews lasted between 45 and 90 min. The participants were from different regions of Norway. The interviews were conducted in three different forums to accommodate the participants and ensure that they were able to participate in the study. Two interviews were conducted in a hospital treatment office, one interview in an office at the University of Oslo (masked for review), and three interviews were conducted digitally. A security-approved digital forum called “Join Service—Norsk Helsenett” was used. The digital conversations were perceived as a good solution, with the participants also reporting that it felt safe. We experienced a similar degree of openness and emotional expression among the participants who participated digitally and physically. The interview guide includes five main categories of questions relating to (1) one’s personal strengths and difficulties, (2) the debut of issues and treatment experiences, (3) the description of a difficult episode (adding that this might relate to loss of control over eating), (4) experiences of the body, and (5) social relations. The questions are formulated as open, more general questions, followed by narrowed questions pertaining to the connection to the body, food, social relations, and treatment experiences. An example of a particular category of questions is: “Could you describe some strong sides to yourself? How would a friend describe you? How would you describe in your own words what is difficult in your life? Is body an issue? In what way?” This structure was chosen to enable the participants to engage more freely, subsequently narrowing the focus on the specific topics of interest.

### 2.4 Data analysis

The data analysis was guided by the reflexive thematic analysis, consisting of six phases (Braun and Clarke, 2006, 2022). The first and second authors independently carried out each phase of the analysis. Then, they met and discussed findings with the third and fourth authors to reach a consensus before entering a new phase. The data material was discussed and presented to all authors in “consensus meetings” (Hill et al., 2005; Hill, 2012) before continuing the analysis. The main purpose of the consensus meetings was to obtain different perspectives and a broader understanding of the research material. These meetings were essential in order to navigate in a coherent and specific direction throughout the research process. The meetings also allowed the researchers to agree on a relevant theoretical context to interpret and discuss the themes. For example, the team agreed on the relevance of attachment theory as the participants shared similar descriptions of their corresponding experiences with early caregivers in relation to how they experienced their illness. This form of researcher triangulation was used to increase the validity of the data material through different perspectives (Fine, 2013).

In the first phase of the analysis, we read and reread the transcripts and wrote individual reflections in a notebook. Interesting details were discussed following each interview (“self-reflective journaling”; Levitt et al., 2017). Based on these reflections and the discussion in a consensus meeting, three analytic questions related to the research questions were prepared. These aimed to explore important themes in the individual interviews and across the portraits (e.g., How did self-esteem affect her relationship with food and her own body? How does body shame affect how she interprets others’ perceptions of her?)

In the second phase, a systematic examination of the material was conducted to identify “codes.” The codes were extracted by rephrasing the participant’s explicit descriptions into the smallest meaningful units possible (Braun and Clarke, 2006). From a part of a participant’s description “… myself takes up much less space than my body …,” the code “the experience of being smaller than her physical size” was extracted. We selected units that provided relevant information for the analytic questions and that could potentially serve as starting points for developing broader themes. The first two authors coded each transcript individually to assess whether there was consistency in interpretation and application of coding principles across the research team. The codes were cross-checked by a third member of the research team to verify that they were meaningful. Codes were extracted manually into a Word file with selected quotes to include all important information for further analyses, reflecting an inductive approach where the codes are close to the participants’ descriptions (Patton and Patton, 1990). Given the size of our material, manual coding was manageable and preferred to allow the researchers to become familiar with the material, also ensuring that the themes were constructed as close to the participant’s descriptions as possible (look at Table 1 for an example of different levels of the analysis).

**Table 1:** Overview of the hierarchy from quotation to meta theme.

Meta theme	Theme	Sub-theme	Code	Group of code	Quotation
Contempt for body image disturbs the experience of self and others (2)	Detachment from body to avoid contact with emotions and being visible (2.1)	I do not relate to what my body is trying to tell me (2.1.1)	She feels like she stops at her neck, where she does not relate to what the body tries to tell her	Body experience	I realize now that I am quite disconnected from my body. In one way, I feel like I stop here *points to neck*. I do not relate to what my body tries to tell me, send me or needs. (…) I probably have not felt my body since I was very young.

In the third phase, the codes were grouped and explored as potential themes. This process involved several consensus meetings, including all authors, where all potentially interesting themes were carefully discussed, selected, and sorted into potential hierarchies with different levels of themes (see Table 1). It was done through a semantic approach, reflecting the immediate meaning of the data (Patton and Patton, 1990). The codes commenced from what the participants had explicitly said, without looking for anything beyond this. The codes were categorized into extensive groups that share a common meaning and encompass a wide range of categories. This step provided an overview of the relationship between codes, groups of codes, potential themes, and different levels of themes.

The fourth phase involved a review of the tentative themes, selected by what were considered to be the most striking and relevant to the research question. During this phase of analysis, we noticed that the data reflected how the participants experienced their illness closely related to their early childhood experiences and influenced their experiences and interpretations of relations as adults. The second observation was reflected in how the participants acted and interpreted in interaction with the researchers during the interview. This inspired the further development and organization of themes. It became important to explore the themes’ internal homogeneity by examining whether data within each theme agree in a meaningful way (Braun and Clarke, 2006). This stage focused on whether each theme described a large enough phenomenon and whether the themes were defined and different rather than overlapping (Patton and Patton, 1990).

In the fifth phase, the themes were named and organized into a hierarchy, where the essence of each theme was defined and the messages conveyed were clarified (Braun and Clarke, 2006). The quotes were connected to the themes with the intention of keeping the themes close to experience. A challenge was to develop titles that were concise and striking and that immediately gave the reader an idea of what the topic was about (Braun and Clarke, 2006). Another challenge was to determine whether something should be a sub-theme or theme, or whether it should represent something as overarching as a meta-theme. In the end, a hierarchy capturing all the data that we desired to include was developed (see Table 1).

The sixth and final phase dealt with printing of the analyses. Although some statements May appear similar, they have different meanings. It was important to select quotes that conveyed the message effectively. It was ensured that the narrative of the analysis did not repeat the quotes but instead added something additional over and above linking the quotes to the research question.

### 2.5 Trustworthiness

Consistent with Hill’s (2012) recommendations, the research team implemented various measures to enhance the trustworthiness of both the research process and its outcomes. Initially, attention was given to fostering reflexivity within the research team. Since the last author participated in group treatment, the research team actively engaged in discussions regarding reflexivity, thereby scrutinizing biases and assumptions related to the data. To increase the reflexivity, associations, and reflections were recorded throughout the research process in a reflexive diary according to the term “self-reflective journaling” (Levitt et al., 2017). In the reflexive diary, each researcher documented immediate thoughts following the interviews, in relation to the participant’s responses and the researcher’s experience of the dialog, and how that could relate to the questions of interest. Surprising details as well as details that were more in line with initial expectations were documented. The discussion of the reflexive diaries, especially with regard to reflections on the dialog between the researchers and the participants, piqued the relevance of including the perspective of metallization. On the other hand, it influenced our focus toward attachment theory, which was not discussed prior to conducting the interviews. In preparing the project, we became aware of the challenges faced by meeting a group that experiences a large degree of body shaming. As female psychology students from Western backgrounds and with slim bodies, we could potentially trigger body shame, which could influence their descriptions of how it feels to live in their bodies. Our awareness could help limit the influence when analyzing and presenting the results. To reduce the risk of bias and strengthen the trustworthiness of the results from the analyses, we worked according to the consensual qualitative research (CQR) framework (Hill, 2012).

### 2.6 Ethics statement

The research project was submitted for approval by the Regional Committees for Medical and Health Research Ethics (Project No. REK 221561) and the Norwegian Centre for Research Data (NSD), where all data were handled in the Service for Sensitive Data (TSD). All applications were approved prior to the recruitment process. In-depth interviews conducted with this group involve ethical dilemmas. Measures to minimize the potential disadvantages of conducting this research project were assessed and applied before, during, and after the data collection. The researchers requested immediate trust from the participants, requiring detailed descriptions of sensitive topics. Before conducting the interview, the researchers discussed how the participants might associate the research project with the trust built during the treatment process. In order to distinguish our research project from the treatment, the participants were asked to participate in the project on the last day of their treatment stay through verbal and written consent, and the interviews were conducted 3 months after admission. During several recruitment and data collection phases, participants were informed that participation was voluntary, that they were free to withdraw at any time without providing a reason, and that their choice would not influence future treatment possibilities at the treatment facility. In order to avoid obtaining excessive information about the participants beyond what is necessary to explore the project’s research questions, a semi-structured interview guide was developed. The participants were encouraged to ask questions or refrain from answering them when asked about sensitive topics. For any inquiries, the contact information for the members of the research team was included in the contract. It was ensured that the participants had access to their general practitioner in the event of any mental health concerns following the interviews, as the topics discussed could provoke reactions. The participants May see value in making this research project possible, with the aim of gaining a deeper understanding of the psychopathology of BED through patients’ own experiences and potentially contributing to acknowledge BED as an ED eligible for treatment. Even though the research aims to provide an authentic picture of these women’s illness, details that would make identification possible, outside of and within the group of participants, were excluded. Given the ethical considerations discussed above, the research team concluded that the potential advantages of conducting this research outweighed the challenges discussed above.

## 3 Results

The analyses of the data resulted in three meta-themes: (1) “Relational challenges and feelings could not be talked about at home,” (2) “Contempt for body image disturbs the experience of self and others,” and (3) “Their body has not been a theme in previous treatment.” Each meta-theme consists of two themes with two subthemes (see Table 2). This classification depicts a story of the disorder in terms of development, current condition, and experiences with prior treatment. In light of our research questions, we are going to present our results with selected quotes from the participants: (1) How do patients with BED experience their suffering? (2) How do patients with BED give meaning to their body and their experience of themselves in social relationships?

**Table 2:** Overview of meta-themes, themes and sub-themes.

Meta theme	Theme	Sub-theme
Relational challenges and feelings could not be talked about at home (1)	Not talking about the problem led to an experience of guilt (1.1)	Nobody talked about why I needed to overeat (1.1.1)
		I had to “pull myself together” (1.1.2)
	A preoccupation with food and weight in the family (1.2)	I became even more preoccupied with my own body when my parents commented on it (1.2.1)
		I have felt that there is something wrong with me—it has been a lot to deal with (1.2.2)
Contempt for body image disturbs the experience of self and others (2)	Detachment from body to avoid contact with emotions and being visible (2.1)	I do not relate to what my body is trying to tell me (2.1.1)
		To disconnect from my feelings and body protects me (2.1.2)
	Self-esteem is linked to weight and food intake (2.2)	Feeling small on the inside and big on the outside (2.2.1)
		It is difficult that somebody likes something I despise (2.2.2)
Their body has not been a theme in previous treatment (3)	It feels like a huge rejection that the body is a non-topic (3.1)	I feel that therapists think my body is a scary theme to discuss (3.1.1)
		There is a big gap between what the treatment works toward and what I want (3.1.2)
	Binge eating is seen more as a medical than a psychological problem (3.2)	When my binge eating disorder is not recognized as an eating disorder, it prevents me from understanding my own mental health condition (3.2.1)
		I cannot go to the doctor without obesity being mentioned, it feels like the society thinks I’m lazy (3.2.2)

### 3.1 Meta-theme 1: relational challenges and feelings could not be talked about at home

The first meta-theme describes how the participants connect their first memories of binge eating with how the family related to food, body, and emotions.

The first theme, “Not talking about the problem led to an experience of guilt” (1.1), reflects the participants’ first memories of overeating and reflecting how emotions were not a topic in the family. All six participants felt that they were not understood and that food was not a way to regulate emotions. The food became a way to cope with emotions, recalling a sense of guilt for overeating and living in a large body. They describe how they had to deal with their binge eating on their own (1.1.1):

*My parents did not realise that anything was wrong… They just said that I wasn’t listening and it did not help with the consequences. I was impossible, I was breaking the rules. They did not take the hint, that this is not normal, and that we should seek help. I did not realise that my relationship with food and body had anything to do with my emotions back then. I’ve never been the type to talk to my parents about emotional things. (Frida).*

Three participants recollect stories in which they attempted to open up about their binge eating habits but were met with the expectation to handle it on their own (1.1.2). Dina describes to us how she tried to speak to her mother about her binge eating:

*I guess I tried to bring it up and was scared and stuff, but my mum got really upset, and would continue feeding me instead. I should not care, I picked up the signals quite quickly that I just have to deal with this on my own somehow. (Dina).*

She seemed to interpret the signals from her mother as saying that difficult experiences should not be discussed. It May serve as an illustration of a demand that several of the participants experienced at an early age, explicitly by using food as problem solving, or through dismissed attempts to open up about difficult experiences.

In the second theme, “A preoccupation with food and weight in the family” (1.2), all six participants describe a prominent focus on food and weight in the family. They reflect on how comments on weight and food intake created a preoccupation with their own body from an early age (1.2.1):

*I do not think it was me who thought I had to lose weight when I was a child, but I got it from my parents (…) Then I became very aware that I had to lose weight to be able to eat with others. (Anna).*

She felt that the regulation of food intake left her feeling like her body should have been smaller. The participants describe how eating became a way of dealing with the discomfort around the family’s preoccupation with their bodies. They depict an experience of being larger than others as synonymous with being flawed (1.2.2):

*I have to fix something, or I have to be different to be good enough (…) Then … I think I started to feel ashamed. Over my body. There was something wrong with it the way I looked, I wasn’t supposed to be like that (…) Because it was clear. I assumed I took up a lot of space or I was too big and everyone wanted me to get smaller. (…) It has been a lot to bear. It is only me that got myself in this body. (Britty).*

A feeling of guilt over taking up too much space and a perception that the overweight was their fault gave me the need to change in order to be good enough. The descriptions pertain to both research questions, with a sense of guilt as a central part of their disorder and a description of their body size as synonymous with faulty functioning.

### 3.2 Meta-theme 2: contempt for body image disturbs the experience of self and others

The second meta-theme explores how participants experience their bodies and how body contempt affects the way they perceive themselves and others.

In the first theme, “Detachment from body to avoid contact with emotions and being visible” (2.1), four participants describe how they experience difficulty sensing their own body and emotions, resulting in shutting off their body’s signals and personal needs from a very young age (2.1.1):

I realise that I am quite disconnected from my body. In one way, I feel like I stop here *points to neck*. I do not relate to what my body tries to tell me, send me or needs. (…) I probably have not felt my body since I was very young. (Frida).

Frida’s sense that her body stops at the neck resonates with several of the other participants. Celina also describes how turning off her body served as a coping mechanism for her challenging relationship with her own body and the feeling of being worthless.

*I had zero contact with my body because I found it so difficult to relate to myself, how I was doing, my own body. I think it’s because I feel like I have zero value, I’m not worth anything. (Celine).*

Three participants perceive the lack of body contact in connection with the experience of feeling less valuable, which affects their relationship with themselves and others. On the other hand, cutting off feelings and body discomfort has helped them function in society (2.1.2):

*It has made me able to walk out the door, go to work. And made me manage to get an education and get a job. If I had taken the dissatisfaction in, and actually listened to all the times I did not want to do things or all those times I did not feel like meeting anyone, I would not have made it out the door! It has had an important function (…) because I think that it is a strength as well, that I have managed to cut off my body’s signals. (Celine).*

Food becomes a means to turn off emotions and disconnect from the body. Three participants describe the food as having a calming effect, helping to reduce self-criticism and stress:

*It’s just a rest… then I get tired and fall asleep (…) it’s like when you take a Oxazepam or two before an exam, and you are really, really nervous and you take those pills, you just ‘wosh’, it goes away, you get a bit numb. (Elena).*

The second theme, “Self-esteem is linked to weight and food intake” (2.2), emphasizes how all six participants feel shame about overeating and how food intake and weight are related to their self-esteem. An experience of ‘being big’ becomes synonymous with being useless in their own eyes and the eyes of others. Three participants describe a feeling of being ‘smaller’ than what their body is physically (2.2.1):

*There is a lot of space in my body. I think I am smaller than what I am physically.*

*I take up much less space than what my body does. I find it difficult being in a room or in the car or anywhere, and experiencing that I am so big. I feel very small and so big at the same time. (Anna).*

Anna describes discomfort with being in a large body and a strong desire not to be visible. An experience of their worth being judged by their body size, linking their self-esteem to their body and relationship with food. The feeling of being judged by their body weight makes it difficult to relate to others. Comments from others regarding their bodies appear to be internalized as their truths. They illustrate a parity between the thoughts of others and their perceptions of their body, which they perceive as encompassing their entire identity (2.2.2). This complicates intimate relationships.

*And because I did not like my body, I thought that no one else did (…) I think people that I have dated think that it’s difficult to be let in. I have managed to screw it up every single time because I do not believe that someone likes me… so when they say positive things I tend to say “you lie, you are not telling the truth, stop saying that,” and then I create a conflict for the relationship to end. Then I will not have to accept that someone might like me and do not have to show myself as a vulnerable person. I reject them first because then I have the control and that is much more comfortable! So I’ve lost quite a few relationships. I have. (Celine).*

The descriptions express how they experience their suffering and give meaning to their body and their experience of self in social relationships. Body contempt makes it difficult to let someone in and believe that other people May like something they despise, complicating intimacy and physical touch. To get through everyday life, the solution is to disconnect the body to avoid body contempt and challenging emotions.

### 3.3 Meta-theme 3: their body has not been a theme in previous treatment

The third meta-theme involves the participant’s experiences of being met by the healthcare system with a BED.

The first theme, “It feels like a huge rejection that the body is a non-topic” (3.1), captures how all six participants describe an experience of a healthcare system where body experience and cause of binge eating have not been thematised before attending treatment. They express how working on body relations is perceived as an important factor in achieving recovery and the frustration of not getting their needs fulfilled in treatment (3.1.1):

*And I have said it all along, until I get to work on the relationship with my body, I will not recover. Because it has been such a big problem for me. (Elena).*

The silence about their bodies in therapy is experienced as a rejection of needs, symbolizing a fear of making their bodies a topic in therapy. They express a big gap between what the treatment conveys and what they want (3.1.2), where the body as a topic becomes more difficult for therapists to bring up the bigger they get, while the need to talk about it increases with weight gain.

*Very few people talk about the body! It is becoming more and more off-topic. The bigger you get, the more off-topic it becomes. And the bigger you get, the more you need to talk about it, and then you are more rejected or belittled or… try to pretend that the body is not important to you, but it is far too important. So there is such a big gap between what they are trying to convey and what I want. (Elena).*

Elena reflects on why her body was a non-topic in therapy:

*When we have been in therapy, the therapists have had a slim body. Is that why they think we cannot talk about it? Because they have a thin body? If they cannot stand that we are fat, I think it is very difficult to have them as therapists! (…) I feel like they thought it was so scary to talk about that they avoided the topic until the last second. (Elena).*

There seems to be a gap between the interests of the therapist and the participants and a feeling of rejection of an important need.

The second theme, “Binge eating is seen more as a medical than a psychological problem” (3.2), highlights how three participants describe that their BED has been overlooked as a mental illness in the healthcare system before they attended treatment. The participants talk about their experiences with the healthcare system, where their problems fall outside the ED category, preventing them from getting the help they need. Their BED not being recognized as an ED prevents them from understanding their disease (3.2.1):

*When I say it out loud to you now that I have an eating disorder, I get embarrassed. When people hear the word eating disorder, they imagine an anorexic person, that’s the image that pops up in people’s minds, making confusion around it. I’m not wanted. Nobody talks about it. Even my doctor after I got out of inpatient treatment for eating disorders. There’s zero knowledge out there, so it is very hard to handle. (Dina).*

They tell us how they cannot go to the doctor without obesity being mentioned, leaving them feeling like society thinks they are lazy (3.2.2). Being met by a healthcare worker with a focus on obesity and interventions for weight loss gives a feeling of guilt.

*I was just told that I had to eat less and exercise more, it depends on willpower. I thought that it is probably like that then (…) I think the most important thing is just to shed light on the fact that this is a disease. It is so undermined in society today, it is so little illuminated. Before I believe it, the average person does not know what a binge eating disorder is and what it does to the lives of people. We are just lazy. (Frida).*

Frida describes an experience of how not being identified as having an ED raises the threshold to seek help, giving valuable insight into how they experience living with a BED and living in a big body. A somatic approach in healthcare with a lack of actualization of body experience in psychotherapy seems to prevent the participants from getting what they need.

## 4 Discussion

This qualitative study aimed to gain a deeper understanding of the psychopathology of BED, by exploring the patients’ meanings related to the disorder and the role of the body in the treatment of BED. The three meta-themes indicate that the emotional climate and food culture within the family are connected to the development of binge eating. It also indicates that body contempt affects self-experience and relationships, where silence about the body in therapy and a focus on obesity in the healthcare system can be experienced as a rejection in the therapeutic alliance and prevent the understanding of BED as a mental disorder.

The descriptions in the first meta-theme reflect how binge eating May become a learned response to deal with emotions alone when difficult experiences could not be discussed in their family of upbringing. This notion is further illuminated by the portrayal of food as a secure attachment figure, a concept supported by a study highlighting the attachment qualities inherent in the patient’s relationship with their ED (Mantilla et al., 2018). Binge eating can lead to a brief reduction in negative effects, potentially mimicking the sense of security and a “safe haven” (Leehr et al., 2015). This coincides with knowledge from developmental psychology that underlines the importance of how the caregiver accepts, accommodates, and modifies the experiences and feelings of their child to help them process unbearable and overwhelming experiences (Thapar et al., 2015; Bion, 1965). From a perspective of metallization and emotion regulation, it is emphasized that when a child becomes left to themselves with too many difficult experiences, the child can establish an experience of not being understood, accommodated, or seen (Fonagy et al., 2002). When a child experiences their parents as emotionally unavailable, it is natural for the child to seek other ways of finding security. Food May become a tool for finding security in the face of overwhelming emotions. This May be understood as a separation trauma that can trigger a response of withdrawal, also described by Winnicott (1974) as a defensive organization. In fear of not getting their feelings accommodated by their parents, food can be integrated as a new attachment figure.

Equally important is the second meta-theme, “body contempt disturbs the experience of self and others,” where the participants describe how shutting down the body signals becomes the solution to turn off self-criticism and stress around the fear of being assessed by themselves and others. Several participants associate their binge eating with the feeling of being less valuable than others. These findings are supported by research that shows that among people with an ED, a high degree of self-criticism leads to increased shame, which contributes to more disturbed eating (Kelly and Carter, 2013). Body shame and self-criticism in the form of disgust and self-hatred have been shown to explain up to 32% of binge eating behavior (Duarte et al., 2014). Binge eating can be both an attempt at self-care and a form of self-neglect. Research on self-compassion in the field of ED focusses on how changes will occur when patients develop the ability to take care of a sense of self they can integrate and represent, which people with an insecure attachment style can find difficult (Gilbert, 2009). The participants highlight how they feel small on the inside while feeling large on the outside—an experience of taking up a large physical space in a room but wanting to be invisible. They describe how food intake and weight are closely related to their self-esteem.

The findings in our study coincide with a large overview reviewing 31 studies, which suggests several potential mechanisms where shame underlies binge eating symptoms (O’Loghlen et al., 2022). If we consider that ED possesses attachment qualities, one hypothesis is that characteristics typical of an ED May become internalized, impacting one’s self-image. This May elucidate how symptoms of ED correlate with self-neglect, self-criticism, self-hatred, and self-control (Mantilla et al., 2018). When the mechanisms are integrated with the self-image, it will affect the experience of the self and others. “Feeling small inside” can be translated to low self-esteem and a high degree of experienced shame. Self-esteem is about the ability to have a nuanced sense of self and, in addition, give what you feel a dignified place about other people (Øiestad, 2009). The feeling of shame can often be experienced as an urge to disappear and as not only having done something wrong but also *being* wrong (Øiestad, 2009). Shame and low self-esteem can linger together, where feelings of shame are experienced so strongly that they undermine self-esteem. For the participants, the experience of being large seems to be synonymous with being less valuable, in their own and other people’s eyes. They relate their feelings to their body in a concrete way in the absence of the ability to integrate the feelings of shame and fear of not being loved and tolerated. The findings also indicate how strong self-criticism has created the need to binge eat to block out the sensation of body contempt and the feeling of being less valuable, a circle of reinforcement of shame, and the need to binge eat as a regulatory mechanism.

In the third meta-theme, the participants describe experiences where obesity becomes the main topic at every doctor’s appointment. At the same time, they experience silence about their body experiences in psychotherapy. The participants are left with the experience of not getting what they need in treatment, in the form of a lack of curiosity related to the cause of the binge eating and how it feels to be in their body. A study that investigated stigmatizing attitudes in the general population toward, e.g., patients with BED, discovered attitudes where BED is associated with a lack of self-discipline (Ebneter and Latner, 2013). These attitudes are recognized in the participants’ experiences and coincide with findings from the study of Riise (2021), where the participants had to prepare well before every doctor’s appointment to avoid obesity becoming the main topic. In the same way that the family’s thematisation of the participants’ obesity has the opposite effect, a focus on obesity in the healthcare system can increase the urge to binge eat. This can be understood as a repetition of the experience of not being understood by early attachment figures (Landmark and Stänicke, 2016). A lack of curiosity about the cause of binge eating is relieved by an encounter with the healthcare system. The weight stigma experienced by the group will affect the patient’s belief in and ability to benefit from health-related advice (Chakravorty, 2021). When health workers induce fat shaming in the patient, body contempt will increase and create an increased need for overeating as a regulatory mechanism for body contempt. This is substantiated by a new overview study suggesting a cyclical relationship between body shame and binge eating symptoms (O’Loghlen et al., 2022). The belittling statements, such as “They said that I had to eat less and exercise more, it depends on willpower,” reflect attitudes in the healthcare system that BED involves little self-discipline and is less severe and complex than other EDs (Reas, 2017). It creates shame for not mastering something as basic as food, which is reinforced by the fact that their loss of control around food does not match their knowledge of food and high functioning in other areas of life. In our study, the participants describe how society only believes that their obesity stems from laziness and little knowledge about nutrition. The patient is left with a lack of understanding of their condition, guilt for feeling too large, and a greater need to binge eat. These findings coincide with a study investigating how attitudes among healthcare workers affect prognosis (Puhl et al., 2014). The results showed that the prognosis is considered to be worse for the patients who meet health workers who discriminate based on body weight, increasing the likelihood of attributing excess weight to behavioral causes and expressing more negative attitudes and greater frustration in treating obese patients. Hence, a somatic approach to the treatment of BED with a main focus on obesity might have negative effects on health, both somatically and psychologically.

The participants also emphasize how larger bodies, as a non-topic in treatment, feel like a big rejection. This experience could be understood as a continuation of established attachment responses. The participants express how they struggle with openness in relationships and miss transparency and honesty in therapy when thoughts about their bodies are so present and emotionally charged. The therapist’s silence can signal that something is unsafe to talk about, where difficult feelings related to their own body cannot be tolerated. Body as a non-topic can be understood as a repetition of the rejection experienced facing parents’ silence about difficult feelings. ED is associated with an avoidant attachment style (O’Shaughnessy and Dallos, 2009; Ty and Francis, 2013). One strategy for patients with ED May be to avoid paying attention to topics that arouse attachment-related feelings toward significant others (Edelstein, 2006). In fear of rejection, the participants themselves describe how they close themselves off, distance themselves, or reject others. In the therapy room, patients can have attachment responses like showing little emotional expression, and the therapist May experience this as distance, which May even arouse boredom (Bateman and Fonagy, 2013). In light of attachment theory, the patient May bring resistance and closedness as an attachment response for protection in the therapy room. If the therapist gives in to the resistance, the patient relives the experienced silence from early attachment figures, which prevents the patient from being able to express themselves about the relationship with their body.

A robust therapeutic alliance stands out as one of the foremost pivotal factors in a therapy conducive to change, characterized by mutual agreement between the patient and the therapist regarding the therapy’s focal point (Bordin, 1979; Wampold, 2015). Research has shown that insecure attachment is associated with a weaker therapeutic alliance (Diener and Monroe, 2011), which suggests that people with insecure attachment May have a vulnerability that challenges the therapeutic work. The participants bring with them an insecurity feeling about new relationships. Their insecure attachment style can be passed on to the therapist, who assumes a caring role, which can be understood as a way to maintain psychological equilibrium. In the face of new relationships, they will maintain psychological closeness to their internalized significant attachment figures. The familiar response is experienced as predictable and safe even if the consequences for new social relationships are negative (Critchfield and Benjamin, 2008). The experienced rejection expressed by the participants can on one side be understood as a refusal from the therapist, and on the other side be understood in the light of repeated attachment responses. These findings reflect Wampold’s (2015) theoretical understanding of effective factors in psychotherapy. In his contextual model, positive outcomes depend on the therapeutic alliance (the therapy relationship), agreement on the therapeutic approach, and an appropriate understanding of the disorder one is trying to treat. The latter seems to have been lacking in the participant’s previous treatment experiences. They describe being met by representatives from a healthcare system who lack knowledge about their condition and suffering, lacking what Wampold’s model (2015) describes as essential to creating effective therapeutic outcomes. This May also explain why the participants never talked about their relationships with their bodies, despite many years in therapy.

## 5 Strengths and limitations

In the following, the strengths and limitations of the study will be discussed in the context of the validity and generalizability of qualitative research. The sample of this research project is selective and small. It is acknowledged that the selective, partial and context-dependent perceptions of the researchers influence the choices made from an idea to the analysis and discussion of results (Stiles, 1993). In line with qualitative research, this study aims to develop theoretical themes based on the participants´ descriptions and not to generalize the data. The themes that have been developed can be discussed according to results from studies with other samples and contexts (Levitt et al., 2017). With this in mind, it could cause issues for the group studied if the information from this qualitative project is generalized. The study’s sample consists of women, and the symptoms for men with EDs May differ in the way they are expressed (Mitchison and Mond, 2015). There will be gender differences in the way people with BED attach meaning to their binge eating and their perception of their bodies. One study suggests that binge eating is more associated with the male roles, which May influence how symptoms are reported (Carey et al., 2017). Despite possible gender differences, experience-based concepts from our study are still discussed with findings from other studies, in line with analytical generalizability in qualitative studies (Kvale et al., 2015). The study’s sample also represents a group of women with BED who have sought out and completed treatment. The sample thus differs from the normal population and persons struggling with binge eating who have not received treatment. Since the group has gone through the same treatment, they May have changed the way they understand and express themselves about their disorder. The treatment can have an impact on which topics they present to us and which concepts they use to attach meaning to their suffering, as they are socialized into a common treatment model (see Sample and selection section). After reading through and analyzing transcripts from interviews, the themes that emerged in the interviews differ to a large extent from the themes brought up during the treatment stay. Especially when it comes to topics related to attachment, their body, and shame in family relationships and therapy. The interviews happened 3 months after the end of treatment, which May have contributed to distinguishing the study from the treatment, strengthening the validity of the findings. In addition, there were more common features than expected across the interviews, even if the topics were new. Aside from limitations related to the study’s sample, it should also be addressed that the theoretical framework of this study has some limitations. It is still missing a wider scientific material of knowledge on BED. There is also a small amount of qualitative research on BED. This study is therefore largely based on research with samples of anorexia, bulimia and other specified ED. Caution should be exhibited if knowledge from this project is used as a knowledge basis for the development of research or treatment on BED specifically. As accounted for in the introduction, it is common to shift from one ED diagnosis to another through a course of illness, which indicates a common vulnerability, where different life events influence how the illness is expressed (Ward et al., 2000). In addition to common mechanisms in the development of the disorders, independently of the type of ED, other common factors are the preoccupation with food, body and weight. This taken into account, the research team has considered research on ED in general as representative of this project’s study.

### 5.1 Clinical and scientific implications

The results of this study have implications for the treatment of and further research on BED. The dissemination of treatment to people with EDs is deficient. Delayed treatment or failure to seek treatment applies to 80–94% of people with bulimia or BED (Rosenvinge and Pettersen, 2015). For comparison, the figure is 50% for people with anorexia. Shame, lack of insight into the disease, and resistance to change are factors that can partially explain these conditions (Rosenvinge and Pettersen, 2015). Lack of treatment-seeking behavior can also be related to the fact that those who struggle with overeating know that evidence-based treatment is not available to them. This constitutes an invitation to the healthcare system and therapists to implement this in clinical practice. The participants emphasize the importance of working with self-limitation in the form of being able to mentally separate their thoughts and feelings from others’ thoughts and feelings. It seems to be important that the therapist shows confidence and commitment in therapy (Gilbert, 2009), giving the patient the security they have not experienced before, which is difficult to give themselves. An openness and curiosity facilitate the patient to explore their condition as a counterbalance to prejudice and oversimplified advice that they have previously experienced. The participants highlight the importance of building a bridge between their emotions and their bodies to get in touch with their body signals and experience a greater acceptance and presence in their bodies. Thematisation of body experience in the therapy room can thus help to capture central experiences with body, self and others in BED.

The questions that arise in light of this are: How useful are the established treatment models for EDs as tools for capturing body shame in the patient? Although recent research shows that psychology increasingly sees the body as one potential therapeutic tool, the integration of this knowledge in practice is a time-consuming process (Ogden and Fisher, 2015). Sensorimotor psychotherapy undermines how after-effects of trauma and attachment problems, in the form of overactivation or underactivation of the body, can manifest in body language and how a person perceives bodily sensations (ref). In the light of attachment theory, the therapist can become a corrective experience in the face of body shame. An attachment figure can challenge the patient’s perception of themselves and their body as disgusting.

The participants convey a need for bodily experience to be thematised in a room with a therapist who dares to engage in conversations about their bodies. In the treatment of BED, integrating different treatment approaches might be appropriate, as already established treatment models for EDs do not provide sufficient effect. Summarizing the implications of the results for clinical practice and the research field, there is a need for more qualitative studies at BED and more specifically on the effect of treatment models that have a focus on body experience in therapy. It will be important to examine BED in the context of empirical models that focus on attachment-related processes such as emotion regulation, metallization and affect integration in the treatment of BED. Recommendations for further studies involve investigating how an open and unafraid therapist can contribute to making a safe space for talking about body shame and how they experience living in a big body, and investigating how talking about and changing their body experience in therapy can contribute to recovery.

## 6 Conclusion

The findings of this study emphasize body experience as a central part of the disorder, where thematisation of the body seems to be an essential part of treatment to achieve change. It contributes to the discussion about how the patients with BED experiences in the face of the healthcare system can experience a repetition of perceived rejection as a child, in the form of a lack of curiosity about the cause of the overeating and a preoccupation with food and weight. It elaborates on the role of body shame in BED and the implications that body experience has for the treatment of BED. The study explores established knowledge around attachment, emotion regulation, and metallization as important aspects in the treatment of BED. It undermines the importance of thematising the patient’s body experience in the therapy room, breaking with previous experiences of the body as a non-topic. This makes the therapist an attachment figure who can create a corrective experience. The findings demonstrate the importance of therapeutic work, which focusses on getting in touch with, regulating and representing emotions and separating one’s inner experiences from others’ experiences.

## Data Availability

The original contributions presented in the study are included in the article/supplementary material, further inquiries can be directed to the corresponding author.

## References

[ref1] American Psychiatric Association (2013). Diagnostic and statistical manual of mental disorders: DSM-5. 5th Edn. Arlington, VA: American Psychiatric Association.

[ref2] Atwood M. E. Friedman A. (2020). A systematic review of enhanced cognitive behavioral therapy (CBT-E) for eating disorders. Int. J. Eat. Disord. 53, 311–330. doi: 10.1002/eat.23206 31840285

[ref3] Austin S. B. (2012). A public health approach to eating disorders prevention: it's time for public health professionals to take a seat at the table. BMC Public Health 12:854. doi: 10.1186/1471-2458-12-854 23043459 PMC3519713

[ref4] Bakland M. Rosenvinge J. H. Wynn R. Sundgot-Borgen J. Fostervold Mathisen T. Liabo K.. (2019). Patients' views on a new treatment for bulimia nervosa and binge eating disorder combining physical exercise and dietary therapy (the PED-t). A qualitative study. Eat. Disord. 27, 503–520. doi: 10.1080/10640266.2018.1560847 30664397

[ref5] Bardone-Cone A. M. Alvarez A. Gorlick J. Koller K. A. Thompson K. A. Miller A. J. (2019). Longitudinal follow-up of a comprehensive operationalization of eating disorder recovery: concurrent and predictive validity. Int. J. Eat. Disord. 52, 1052–1057. doi: 10.1002/eat.23128 31291036

[ref6] Bateman A. Fonagy P. (2013). Mentalization-based treatment. Psychoanal. Inq. 33, 595–613. doi: 10.1080/07351690.2013.835170 26157198 PMC4467231

[ref7] Bion W. R. (1965). Transformation. London: Karnac Books.

[ref8] Bordin E. S. (1979). The generalizability of the psychoanalytic concept of the working alliance. Psychotherapy (Chic.) 16, 252–260. doi: 10.1037/h0085885

[ref9] Braun V. Clarke V. (2006). Using thematic analysis in psychology. Qual. Res. Psychol. 3, 77–101. doi: 10.1191/1478088706qp063oa

[ref10] Braun V. Clarke V. (2022). Conceptual and design thinking for thematic analysis. Qual. Psychol. 9, 3–26. doi: 10.1037/qup0000196

[ref11] Bremer M. F. Garnweidner-Holme L. Nesse L. Molin M. (2023). Experiences of living with binge eating disorder and facilitators of recovery processes: a qualitative study. J. Eat. Disord. 11:201. doi: 10.1186/s40337-02300929-2 37964397 PMC10647123

[ref12] Brownley K. A. Berkman N. D. Sedway J. A. Lohr K. N. Bulik C. M. (2007). Binge eating disorder treatment: a systematic review of randomized controlled trials. Int. J. Eat. Disord. 40, 337–348. doi: 10.1002/eat.20370 17370289

[ref13] Brownstone L. M. Mihas P. M. Butler R. Maman S. Peterson C. B. Bulik C. M.. (2021). Lived experiences of subjective binge eating: an inductive thematic analysis. Int. J. Eat. Disord. 54, 2192–2205. doi: 10.1002/eat.23636 34761418

[ref14] Bulik C. M. Sullivan P. F. Kendler K. S. (2002). Medical and psychiatric morbidity in obese women with and without binge eating. Int. J. Eat. Disord. 32, 72–78. doi: 10.1002/eat.10072 12183948

[ref15] Carey J. B. Saules K. K. Carr M. M. (2017). A qualitative analysis of men's experiences of binge eating. Appetite 116, 184–195. doi: 10.1016/j.appet.2017.04.030 28465183

[ref16] Chakravorty T. (2021). Fat shaming is stopping doctors from helping overweight patients here’s what medical students can do about it. BMJ (Online) 375:n2830. doi: 10.1136/bmj.n2830 34789468

[ref17] Critchfield K. L. Benjamin L. S. (2008). Internalized representations of early interpersonal experience and adult relationships: a test of copy process theory in clinical and non-clinical settings. Psychiatry 71, 71–92. doi: 10.1521/psyc.2008.71.1.71 18377207

[ref18] Culbert K. M. Racine S. E. Klump K. L. (2015). Research review: what we have learned about the causes of eating disorders - a synthesis of sociocultural, research review: what we have learned about the causes of eating disorders – a synthesis of sociocultural, psychological, and biological research, psychological, and biological research. J. Child Psychol. Psychiatry 56, 1141–1164. doi: 10.1111/jcpp.12441 26095891

[ref19] Delis D. C. Kaplan E. Kramer J. (2001). Delis Kaplan executive function system. San Antonio: The Psychological Corp.

[ref20] Diener M. J. Monroe J. M. (2011). The relationship between adult attachment style and therapeutic alliance in individual psychotherapy: a meta-analytic review. Psychotherapy (Chic.) 48, 237–248. doi: 10.1037/a0022425 21604902

[ref21] Duarte C. Pinto-Gouveia J. Ferreira C. (2014). Escaping from body image shame and harsh self-criticism: exploration of underlying mechanisms of binge eating. Eat. Behav. 15, 638–643. doi: 10.1016/j.eatbeh.2014.08.025 25248129

[ref22] Ebneter D. S. Latner J. D. (2013). Stigmatizing attitudes differ across mental health disorders: a comparison of stigma across eating disorders, obesity, and major depressive disorder. J. Nerv. Ment. Dis. 201, 281–285. doi: 10.1097/NMD.0b013e318288e23f 23538972

[ref23] Edelstein R. S. (2006). Attachment and emotional memory: investigating the source and extent of avoidant memory impairments. Emotion 6, 340–345. doi: 10.1037/1528-3542.6.2.340 16768567

[ref24] Fairburn C. G. Cooper Z. Doll H. A. Welch S. L. (1999). Risk factors for anorexia nervosa: three integrated case-control comparisons. Arch. Gen. Psychiatry 56, 468–476. doi: 10.1001/archpsyc.56.5.468 10232302

[ref25] Fairburn C. G. Sjøbu A. (2014). Få bukt med overspising: hvorfor overspiser man, og hvordan slutter man med det? Gyldendal akademisk.

[ref26] Fine M. (2013). Echoes of Bedford: a 20-year social psychology memoir on participatory action research hatched behind bars. Am. Psychol. 68, 687–698. doi: 10.1037/a0034359, PMID: 24320653

[ref27] Fonagy P. Gergely G. Jurist E. L. Target M. (2002). Affect regulation, mentalization, and the development of the self. London: Other Press.

[ref28] Gilbert P. (2009). The compassionate mind: A new approach to life challenges. London: Constable and Robinson Ltd.

[ref29] Gilbert P. (2014). The origins and nature of compassion focused therapy. Br. J. Clin. Psychol. 53, 6–41. doi: 10.1111/bjc.12043 24588760

[ref30] Goss K. (2011). The compassionate mind approach to beating overeating: Using compassion focused therapy. London: Robinson.

[ref31] Goss K. Allan S. (2010). Compassion focused therapy for eating disorders. Int. J. Cogn. Ther. 3, 141–158. doi: 10.1521/ijct.2010.3.2.141

[ref32] Goss K. Allan S. (2014). The development and application of compassion-focused therapy for eating disorders (CFT-E). Br. J. Clin. Psychol. 53, 62–77. doi: 10.1111/bjc.12039 24588762

[ref33] Götestam K. G. Agras W. S. (1995). General population-based epidemiological study of eating disorders in Norway. Int. J. Eat. Disord. 18, 119–126. doi: 10.1002/1098-108x(199509)18:2<119::aid-eat2260180203>3.0.co;2-u 7581413

[ref34] Hilbert A. Petroff D. Herpertz S. Pietrowsky R. Tuschen-Caffier B. Vocks S.. (2019). Meta-analysis of the efficacy of psychological and medical treatments for binge-eating disorder. J. Consult. Clin. Psychol. 87, 91–105. doi: 10.1037/ccp0000358 30570304

[ref35] Hill C. E. (Ed.) (2012). Consensual qualitative research: A practical resource for investigating social science phenomena. Washington, DC: American Psychological Association.

[ref36] Hill C. E. Knox S. Thompson B. J. Williams E. N. Hess S. A. Ladany N. (2005). Consensual qualitative research: an update. J. Couns. Psychol. 52, 196–205. doi: 10.1037/0022-0167.52.2.196

[ref37] Kelly A. C. Carter J. C. (2013). Why self-critical patients present with more severe eating disorder pathology: the mediating role of shame. Br. J. Clin. Psychol. 52, 148–161. doi: 10.1111/bjc.12006, PMID: 24215145

[ref38] Kelly A. C. Carter J. C. (2015). Self-compassion training for binge eating disorder: a pilot randomized controlled trial. Psychol. Psychother. Theory Res. Pract. 88, 285–303. doi: 10.1111/papt.12044 25330466

[ref39] Kelly A. C. Tasca G. A. (2016). Within-persons predictors of change during eating disorders treatment: an examination of self-compassion, self-criticism, shame, and eating disorder symptoms. Int. J. Eat. Disord. 49, 716–722. doi: 10.1002/eat.22527 27061929

[ref40] Kelly A. C. Vimalakanthan K. Carter J. C. (2014). Understanding the roles of self-esteem, self-compassion, and fear of self-compassion in eating disorder pathology: an examination of female students and eating disorder patients. Eat. Behav. 15, 388–391. doi: 10.1016/j.eatbeh.2014.04.008, PMID: 25064287

[ref41] Keski-Rahkonen A. (2021). Epidemiology of binge eating disorder: prevalence, course, comorbidity, and risk factors. Curr. Opin. Psychiatry 34, 525–531. doi: 10.1097/YCO.0000000000000750, PMID: 34494972

[ref42] Kessler R. C. Berglund P. A. Chiu W. T. Deitz A. C. Hudson J. I. Shahly V.. (2013). The prevalence and correlates of binge eating disorder in the World Health Organization world mental health surveys. Biol. Psychiatry 73, 904–914. doi: 10.1016/j.biopsych.2012.11.020 23290497 PMC3628997

[ref43] Kvale S. Brinkmann S. Anderssen T. M. Rygge J. (2015). “Det kvalitative forskningsintervju” in Gyldendal akademisk. 3rd ed.

[ref44] Landmark A. F. Stänicke L. I. (2016). “Det uforståelige barnet: om å skape sammenheng mellom den indre og ytre verden” in Hertervig forl., Akademisk.

[ref45] Leehr E. J. Krohmer K. Schag K. Dresler T. Zipfel S. Giel K. E. (2015). Emotion regulation model in binge eating disorder and obesity--a systematic review. Neurosci. Biobehav. Rev. 49, 125–134. doi: 10.1016/j.neubiorev.2014.12.008 25530255

[ref46] Levitt H. M. Motulsky S. L. Wertz F. J. Morrow S. L. Ponterotto J. G. (2017). Recommendations for designing and reviewing qualitative research in psychology:promoting methodological integrity. Qual. Psychol. (Washington, D.C.) 4, 2–22. doi: 10.1037/qup0000082

[ref47] Lewke-Bandara R. S. Thapliyal P. Conti J. Hay P. (2020). It also taught me a lot about myself": a qualitative exploration of how men understand eating disorder recovery. J. Eat. Disord. 8:3. doi: 10.1186/s40337-020-02796 32025303 PMC6996167

[ref48] Lord V. M. Reiboldt W. Gonitzke D. Parker E. Peterson C. (2018). Experiences of recovery in binge-eating disorder: a qualitative approach using online message boards. Eat. Weight Disord. 23, 95–105. doi: 10.1007/s40519-016-0335-z 27796846

[ref49] Mantilla E. F. Clinton D. Birgegård A. (2018). The unsafe haven: eating disorders as attachment relationships. Psychol. Psychother. 92, 379–393. doi: 10.1111/papt.12184, PMID: 29781234

[ref50] McLeod J. (2011). Qualitative research in counselling and psychotherapy. 2nd Edn. London: Sage.

[ref51] Mitchison D. Mond J. (2015). Epidemiology of eating disorders, eating disordered behaviour, and body image disturbance in males: a narrative review. J. Eat. Disord. 3:20. doi: 10.1186/s40337-015-0058-y 27408719 PMC4940910

[ref52] Moghimi E. Davis C. Bonder R. Knyahnytska Y. Quilty L. (2022). Exploring women's experiences of treatment for binge eating disorder: methylphenidate vs. cognitive behavioural therapy. Prog. Neuro-Psychopharmacol. Biol. Psychiatry 114:110492. doi: 10.1016/j.pnpbp.2021.110492 34863926

[ref53] National Institute for Health and Care Excellence. (2017). Eating disorders: Recognition andTreatment [NICE guideline no. 69]. Available at: https://www.nice.org.uk/guidance/ng69.

[ref54] O’Loghlen E. Grant S. Galligan R. (2022). Shame and binge eating pathology: a systematic review. Clin. Psychol. Psychother. 29, 147–163. doi: 10.1002/cpp.2615 34010473

[ref55] O’Shaughnessy R. Dallos R. (2009). Attachment research and eating disorders: a review of the literature. Clin. Child Psychol. Psychiatry 14, 559–574. doi: 10.1177/1359104509339082 19759074

[ref56] Ogden P. Fisher J. (2015). Sensorimotor psychotherapy: Interventions for trauma and attachment. Hierro D. Del Hierro A. Del, Eds. New York: W W Norton & Co.

[ref57] Øiestad G. (2009). Selvfølelsen. Oslo: Gyldendal Norsk Forlag A/S isbn:978-82-05-38476-7.

[ref58] Patton M. Q. Patton M. Q. (1990). Qualitative evaluation and research methods. 2nd Edn. Newbury Park, CA: Sage.

[ref59] Perelman H. Gilbert K. Grilo C. M. Lydecker J. A. (2023). Loss of control in binge eating disorder: fear and resignation. Int. J. Eat. Disord. 56, 1199–1206. doi: 10.1002/eat.23929, PMID: 36920120 PMC10247475

[ref60] Puhl R. M. Latner J. D. King K. M. Luedicke J. (2014). Weight bias among professionals treating eating disorders: attitudes about treatment and perceived patient outcomes. Int. J. Eat. Disord. 47, 65–75. doi: 10.1002/eat.22186 24038385

[ref61] Reas D. L. (2017). Public and healthcare Professionals' knowledge and attitudes toward binge eating disorder: a narrative review. Nutrients 9:1267. doi: 10.3390/nu9111267, PMID: 29160843 PMC5707739

[ref62] Riise J. (2021). Å møte helsevesenet med overspisingslidelse - En kvalitativ studie av hvordan personer med overspisingslidelse forstår sine behov i behandling.

[ref63] Rørtveit K. Furnes PhD B. Dysvik PhD E. Ueland PhD V. (2021). Patients' experience of attending a binge eating group program - qualitative evaluation of a pilot study. SAGE Open Nurs. 7:23779608211026504:237796082110265. doi: 10.1177/23779608211026504 34345676 PMC8283049

[ref64] Rosenvinge J. H. Pettersen G. (2015). Epidemiology of eating disorders part III: social epidemiology and case definitions revisited. Adv. Eat. Disord. (Abingdon) 3, 320–336. doi: 10.1080/21662630.2015.1022197

[ref65] Salvia M. G. Ritholz M. D. Craigen K. L. E. Quatromoni P. A. (2022). Managing type 2 diabetes or prediabetes and binge eating disorder: a qualitative study of patients’ perceptions and lived experiences. J. Eat. Disord. 10:148. doi: 10.1186/s40337-022-00666-y, PMID: 36221145 PMC9554983

[ref66] Salvia M. G. Ritholz M. D. Craigen K. L. E. Quatromoni P. A. (2023). Women's Perceptions of weight stigma and experiences of weight-neutral treatment for binge eating disorder: a qualitative study. EClinicalMedicine, 56, 101811. doi:10.1016/j.eclinm.2023.101811 10.1016/j.eclinm.2022.101811 PMC9816903 36618893

[ref67] Serpell L. Amey R. Kamboj S. K. (2020). The role of self-compassion and self criticism in binge eating behaviour. Appetite 144:104470. doi: 10.1016/j.appet.2019.104470 31586596

[ref630] Thapar A. Pine D. S. Leckman J. F. Scott S. Snowling M. J. Taylor E. (2015). Child and Adolesc Psych. John Wiley and Sons, Ltd.

[ref68] Stiles W. B. (1993). Quality control in qualitative research. Clin. Psychol. Rev. 13, 593–618. doi: 10.1016/0272-7358(93)90048-Q

[ref69] Ty M. Francis A. J. P. (2013). Insecure attachment and disordered eating in women: the mediating processes of social comparison and emotion dysregulation. Eat. Disord. 21, 154–174. doi: 10.1080/10640266.2013.761089 23421698

[ref70] Wampold B. E. (2015). How important are the common factors in psychotherapy? An update. World Psychiatry 14, 270–277. doi: 10.1002/wps.20238 26407772 PMC4592639

[ref71] Ward A. Ramsay R. Treasure J. (2000). Attachment research in eating disorders. Br. J. Med. Psychol. 73, 35–51. doi: 10.1348/000711200160282 10759049

[ref72] Watson H. J. Jangmo A. Smith T. Thornton L. M. von Hausswolff-Juhlin Y. Madhoo M.. (2018). A register-based case-control study of health care utilization and costs in binge-eating disorder. J. Psychosom. Res. 108, 47–53. doi: 10.1016/j.jpsychores.2018.02.011 29602325

[ref74] Wilson G. T. Grilo C. M. Vitousek K. M. (2007). Psychological treatment of eating disorders. Am. Psychol. 62, 199–216. doi: 10.1037/0003-066X.62.3.199 17469898

[ref75] Winnicott D. (1974). Fear of breakdown. Int. Rev. Psycho-Anal. 1, 103–107.

[ref76] World Health Organization (2019). International statistical classification of diseases and related health problems. 11th Edn Available at: https://icd.who.int/. 10.2471/BLT.25.294650 PMC13531092 42683092

